# Moths sense but do not learn flower odors with their proboscis during flower investigation

**DOI:** 10.1242/jeb.242780

**Published:** 2021-09-13

**Authors:** Elisabeth Adam, Bill S. Hansson, Markus Knaden

**Affiliations:** Department of Evolutionary Neuroethology, Max Planck Institute for Chemical Ecology, D-07745Jena, Germany

**Keywords:** *Manduca sexta*, Hawkmoth, Insect olfaction, Flower handling, *Nicotiana attenuata*, Learning

## Abstract

Insect pollinators, such as the tobacco hawkmoth *Manduca sexta*, are known for locating flowers and learning floral odors by using their antennae. A recent study revealed, however, that the tobacco hawkmoth additionally possesses olfactory sensilla at the tip of its proboscis. Here, we asked whether this second ‘nose’ of the hawkmoth is involved in odor learning, similar to the antennae. We first show that *M. sexta* foraging efficiency at *Nicotiana attenuata* flowers increases with experience. This raises the question whether olfactory learning with the proboscis plays a role during flower handling. By rewarding the moths at an artificial flower, we show that, although moths learn an odor easily when they perceive it with their antennae, experiencing the odor just with the proboscis is not sufficient for odor learning. Furthermore, experiencing the odor with the antennae during training does not affect the behavior of the moths when they later detect the learned odor with the proboscis only. Therefore, there seems to be no cross-talk between the antennae and proboscis, and information learnt by the antennae cannot be retrieved by the proboscis.

## Abstract (precis)

**Highlighted Article:** The hawkmoth *Manduca sexta* is able to detect odors with the tip of its tongue: this ‘second nose’ is not used for olfactory learning during flower investigation.

## INTRODUCTION

While foraging, flower-visiting insects have to maximize their energy gain while keeping their energy expenditure at a minimum (Pyke, 1978). This is especially crucial for hovering insects such as hawkmoths, as they are faced with considerable energy costs during flight (Bartholomew and Casey, 1978). Therefore, it is of great advantage to learn reliable resources and avoid flowers that produce no nectar or nectar of low quality, cheating the insect out of a profitable meal. Because primary attractants such as nectar and pollen are often concealed, insects have to rely on other, secondary attractants, e.g. color, shape, flower scent, taste or texture, to locate the floral rewards (Wester and Lunau, 2017). This is particularly important, since not every nectar is scented, which would be a honest signal for the pollinator (Raguso, 2004).

Moths use both flower scent and visual displays as secondary attractants to locate their host flowers (Raguso and Willis, 2002, 2005; Stockl and Kelber, 2019). Depending on their circadian rhythm, the agile hawkmoths are known to assign different relative importance to these stimuli (Balkenius et al., 2006). While the diurnal moth *Macroglossum stellatarum* predominantly reacts to visual stimuli, the nocturnal moth *Deilephila elpenor* is strongly attracted by olfactory stimuli (Balkenius et al., 2006). The crepuscular hawkmoth *Manduca sexta*, in contrast, uses both visual and olfactory cues to locate host flowers (Raguso and Willis, 2002, 2005) and only solely relies on olfactory cues under very dim light conditions (Goyret and Yuan, 2015).

In the wild, *M. sexta* is innately attracted to the floral scent of *Nicotiana attenuata* and *Datura wrightii*, and is known to pollinate flowers that share a similar chemical odor profile (Kessler and Baldwin, 2007; Riffell et al., 2013). Many individual odorants of these flower profiles are sufficient to induce foraging behavior in *M. sexta*, while other odorants are innately neutral (Bisch-Knaden et al., 2018). Yet, the hawkmoth can also learn to follow innately neutral odors of rewarding flowers when it is first lured to these flowers via visual cues (Cook et al., 2020; Riffell et al., 2008, 2013). This ability to associate novel odors with a reward has also been demonstrated in tethered moths, which exhibited a proboscis extension reflex after they had perceived an odor together with a sucrose reward (Daly et al., 2001b; Daly and Smith, 2000). However, in nature, moths not only have to locate the flower but, in a subsequent step, also need to be able to find the nectar within the flower (Goyret and Raguso, 2006). This is no easy feat, as the moth has to hover in front of the flower to access the nectar, a behavior that is energetically quite costly (Bartholomew and Casey, 1978). The involvement of additional sensory channels, including vision and mechanosensation, has been shown to enhance flower handling capabilities: although vision is important during both flower tracking and flower investigation, mechanosensation only seems to play an important role during flower handling itself (Farina et al., 1994; Goyret, 2010; Goyret and Kelber, 2011).

Recently, Haverkamp et al. (2016b) described newly discovered olfactory sensilla at the tip of the moth's proboscis. These sensilla house Orco-positive neurons that are able to detect and process odorants and respond to several of the same floral odorants that are also detected by the moth's antennae (Haverkamp et al., 2016b). The study revealed that this additional olfactory sense at the tip of the proboscis lets the moth investigate an innately attractive odor within the flower. Here, we asked whether the olfactory sensilla at the tip of the hawkmoth's proboscis could have an additional function in olfactory learning. As hawkmoths are known for their fast olfactory learning via their antennae (Cook et al., 2020; Riffell et al., 2008), we argued that learning new olfactory properties of the flower via the proboscis could give the moth an advantage in the identification and handling of consecutive flowers. This would be particularly true if there was cross-talk between the proboscis and antennae, as inserting the proboscis to smell the odor is energetically more costly (as a result of hovering and tracking the flower) than recognizing the odor from afar. Therefore, we investigated whether hawkmoths are able to become more proficient in handling natural flowers that emit an appetitive odor over time and whether they use their sense of smell on the proboscis to learn flower cues. Also, by carefully modifying the test paradigms and working with an artificial flower that allowed us to confine odor to the proboscis only, we explored whether there is indeed cross-talk between the two ‘noses’ of the hawkmoth.

## MATERIALS AND METHODS

### Hawkmoth rearing

*Manduca sexta* (Linnaeus 1763) moths were reared and maintained at the Max Planck Institute for Chemical Ecology (MPI CE). Adult moths were kept in a flight cage (122×42×76 cm) for mating. A *N. attenuata* plant served as a substrate for oviposition, and a hummingbird feeder with artificial flowers provided a 10% sugar solution. Eggs were collected 3 times a week and transferred to small plastic boxes containing an artificial diet for the emerging larvae (artificial diet: 306 g agar, 959 g wheat germ, 932 g corn meal, 506 g soy flour, 499 g casein, 160 g salt mix, 240 g sugar, 33.3 g cholesterol, 80 g ascorbic acid, 40 g sorbic acid, 20 g methyl paraben, 60 ml linseed oil, 200 ml vitamin mix and 12 l water; vitamin mix: 2000 mg nicotinic acid, 1000 mg riboflavin, 467 mg thiamine, 467 mg pyridoxine, 467 mg folic acid, 40 mg biotin and 2 l water). On reaching 2nd instar, larvae were transferred to larger boxes; 5th instar larvae, which stopped feeding, were moved to wooden planks with pre-drilled holes for pupation. Shortly before hatching, male and female pupae were separated and put into egg cartons inside small folding insect cages. Hatched moths were kept in larger flight cages until experimentation. Eggs, larvae and pupating larvae were reared in a climate-controlled chamber at 26°C and 40% humidity and a light:dark cycle of 15 h:9 h. Pupae and adult moths were kept in separate climate chambers for male and female moths at 25°C and 60% humidity during light hours and 23°C and 70% humidity during dark hours on a light:dark cycle of 16.5 h:7.5 h. For all behavioral experiments, 3 day old naive male moths, which had neither fed nor encountered an (artificial) flower before, were used.

### Wind tunnel experiments

All behavioral experiments were conducted in a wind tunnel with laminar air flow (wind tunnel size: 250×94×90 cm; wind speed – charcoal filtered air: 0.4 m s^−1^; temperature: 25°C; humidity 70–75%). Side, top and front cameras were used to record the movement of the moth within the wind tunnel (Logitech HD Webcam C615; ELP HD Digital USB Camera; recording speeds: 30 frames s^−1^). White and red light sources produced low visible light (photosynthetic active radiation, PAR: 0.27 μmol m^−2^ s^−1^). To allow recording of the moth at these low light settings, the infrared filters of the cameras were removed and an infrared light source (not visible to the moth) was used for illumination. Videos were recorded using Noldus Media Recorder 2.5.228 software (Noldus, Wageningen, The Netherlands) to allow the simultaneous recording of different camera angles.

The hawkmoths were kept in individual small mesh cages (14.5 cm diameter and 14.5 cm high) and acclimatized to the wind tunnel conditions in a habituation chamber for at least 1 h prior to testing. In all paradigms, the moths started from a small platform (height: 33.5 cm) positioned centrally at the downwind end of the wind tunnel, about 10 cm from the wind tunnel wall. They were gently nudged with a brush to initiate wing fanning (for 1–2 min). As soon as a moth started to fly, it was allowed to explore the wind tunnel for 5 min. Trials ended after the time had expired or if the moth landed, as the animals generally did not resume flight for several minutes after landing.

### Real flower investigation

**Fig. 1.**
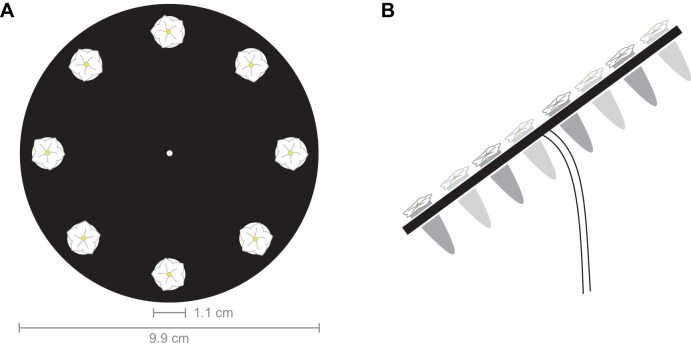
**Real flower array.** (A) Black acrylic disc holding eight Eppendorf tubes with *Nicotiana attenuata* flowers that can be investigated by the moth. Tubes behind the disc provide water to prevent flower desiccation. (B) Side view of the flower array. The disc is held by a bendable rod to allow for an upward angle. Drawings not to scale.

To establish whether learning improves the moths’ success in handling natural flowers and create a baseline learning curve in our wind tunnel set-up, we first tested the moths using flowers of the wild tobacco plant *N. attenuata*. It is known that *M. sexta* improves its flower handling at artificial flowers over time (Goyret and Raguso, 2006). However, we wanted to confirm that this is also the case for natural flowers. As *M. sexta* is known as a major pollinator of *N. attenuata* (Kessler et al., 2010, 2015), and it has been shown that the odor emitted by these flowers increases foraging motivation in the hawkmoth even in the absence of nectar (Haverkamp et al., 2016b), flowers of this plant were chosen for the paradigm. Compared with large flowers of plants such as *D. wrightii* (in which the moth simply has to drop its body and proboscis), the tiny flowers of *N. attenuata* are more difficult to handle (Haverkamp et al., 2016a). Therefore, we expected that learning-based improved flower handling would become more obvious with these rather tricky flowers. A flower array containing eight *N. attenuata* flowers was positioned at the upwind end of the wind tunnel (Fig. 1A). We decided to use a flower array instead of the whole plant to standardize flower angle and to allow for better video recording of the flower manipulation. In the wild, the flowers of *N. attenuata* move through a 140 deg arc from a pendant position (daytime) to an erect position (night-time) to facilitate interaction with the hawkmoth (Haverkamp et al., 2019; Yon et al., 2016, 2017). Therefore, we mounted the disc containing the flowers at a slight upward angle to ease the insertion of the proboscis. The *N. attenuata* flowers contained their natural nectar volumes. No additional sugar water or artificial nectar was added, in order to create as natural a testing situation as possible. The flowers were kept in water-filled 1.5 ml Eppendorf tubes with a hole drilled in the lid to prevent flower desiccation during testing (Fig. 1B). During the trial, the moth could freely investigate all eight flowers with its proboscis. It was counted as ‘moth flown’ if it initiated flight during the trial without settling down immediately afterwards. Moths that touched a flower within the flower array at least once with their proboscis were counted as ‘investigated flower’. To find out whether the moths become more efficient in flower handling, their success at inserting the proboscis into the flower during consecutive flower visits was analyzed.

### Artificial flower design and training apparatus

**Fig. 2.**
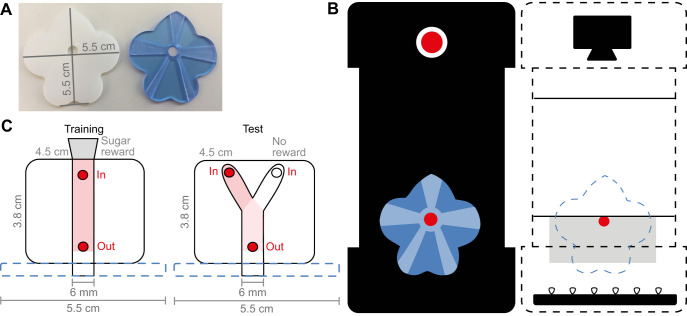
**Proboscis training apparatus.** (A) Artificial flower. Left: white back layer to define flower shape. Right: light blue front layer with high reflective properties and radial recesses. (B) Training apparatus. Left: front view of the outside of the vertical apparatus. The artificial flower (blue) at the front of the training apparatus allows insertion of the proboscis. A clamp holding a small filter paper disc (red disc with white outline) for antennal odor stimulation can be attached to the front of the apparatus in such a way that the moth does not accidently touch it while investigating the flower. Right: front view of the inside of the vertical apparatus. The grey rectangle indicates the space for the exchangeable elements (see C). A video camera (black) records the insertion of the proboscis from above. Infrared LEDs (small triangles) illuminate the proboscis training tube/Y-maze from below. Red circles indicate odor on the filter paper disc and/or in the proboscis training tube. (C) Top view of the exchangeable elements. Left: the proboscis can be inserted in a proboscis training tube through the artificial flower (blue outline). To exclude odor leakage out of the flower, odor is supplied through the valve labelled ‘In’ and removed by vacuum through the valve labelled ‘Out’. The sugar reward (gray trapezium) is provided at the end of the proboscis training tube. Right: again, the proboscis can be inserted into the Y-maze through the artificial flower (blue outline). Odor and clean air (mineral oil control) (red and white circles) can be supplied through the valves labelled ‘In’ and removed through the valve labelled ‘Out’. Drawings not to scale.

To be able to train the moths and let them experience an odor together with a reward, we designed an artificial flower (Fig. 2A) to visually attract the moths to our training apparatus (Fig. 2B). We created a rather large flower compared with *N. attenuata* in order create a supernormal stimulus and visually attract the moths. This was necessary as hawkmoths are less likely to respond to odorless flowers than to flowers emitting odor (Goyret et al., 2007; Raguso and Willis, 2002, 2005). The moths were rewarded with sugar water – in the presence or absence of the given training odor – at this artificial flower. The flower consisted of two layers of laser cut acrylic sheet with five petals and a central opening (flower measurements: 5.5×5.5×3 mm; central opening diameter: 6 mm). A white back layer defined the outline of the flower; a glossy light blue front layer (without UV reflective properties) enhanced the attraction of the flower (Fig. 2A). We chose these materials as our pre-tests with artificial flowers made with different materials/colors/reflective properties had shown that hawkmoths have an innate preference for blue flowers with highly reflective properties outside the UV range, confirming the results of Goyret et al. (2008). Previous studies further showed that hawkmoths prefer radial patterns and that radial folds as well as a slight corolla curvature can improve flower handling efficiency (Campos et al., 2015; Goyret and Raguso, 2006; Kelber, 2002). Therefore, we also added radial recesses leading to the center of the flower (radial recesses: 1 mm). The artificial flower was attached to an apparatus that enabled us to track the movement of the proboscis within the flower, so we could confirm that the moths reached the sugar reward. The apparatus (Fig. 2B) consisted of a top lid containing a video camera (Logitech Webcam 600; recording speed: 30 frames s^−1^; resolution: 640×480 pixels), a middle part acting as a spacer/odor barrier and a bottom part containing an exchangeable element with either a linear proboscis training tube or a Y-maze (Fig. 2C). To be able to record the insertion of the proboscis, an infrared LED panel (Roschwege GmbH, LED puzzle piece, IR 850 nm) was added under the exchangeable element (Fig. 2B). The LEDs were powered at 11.6 V and 0.14 A (Manson switching mode power supply NRP 3630). An opening at the front of the training apparatus (diameter 6 mm) connected the artificial flower to the linear tube/Y-maze inside the apparatus. The training apparatus containing the proboscis training tube had an additional opening at the back (diameter 6 mm) to allow the attachment of a PCR tube with the sugar reward for conditioning (PCR tube: 200 μl, cut in half; sugar reward: 75 μl of 30% sucrose solution; sucrose: CAS 57-50-1). To facilitate the insertion of the proboscis, the training apparatus was mounted at a slight upward angle (+20 deg from the horizontal axis).

### Odor choice paradigm

**Fig. 3.**
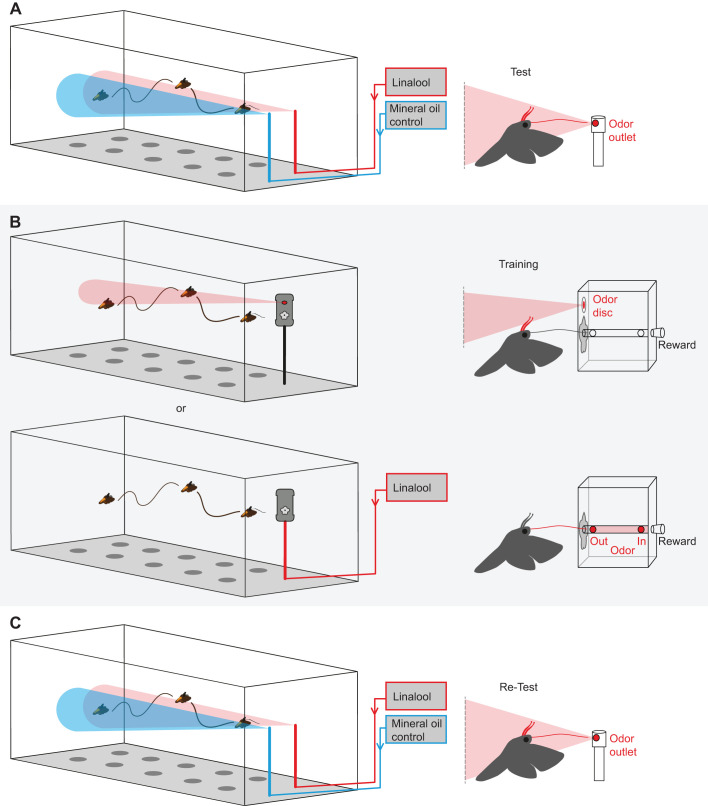
***Manduca* odor choice paradigm.** (A) Moths were tested for innate attraction to training odor in the wind tunnel. One odor valve supplied training odor (linalool, red), the other valve clean air (mineral oil control, blue). (B) During training, odor was either supplied to the antennae (top right) or restricted to the proboscis (bottom right) while a sugar reward was provided. (C) For the re-test, moths were again given a choice between the training odor (linalool) and clean air (mineral oil control). Drawings not to scale.

To investigate whether *M. sexta* moths are able to learn odors that they perceive with their proboscis, we first established the innate valence of the training odor in a choice experiment (Fig. 3A). In brief, individual moths were allowed to explore the wind tunnel and given a choice between two odor sources, one emitting clean air (mineral oil control), the other the training odor. We then measured the time the moths spent investigating both sources. We wanted to select an innately neutral odor (i.e. neither aversive nor attractive), to be able to show olfactory learning. Therefore, we chose linalool (+/−) (CAS 78-70-6), as it had previously been shown to be innately neutral in experiments investigating the antennal olfactory perception of the moth (Bisch-Knaden et al., 2018). Further, it can be perceived by the proboscis of the moth (Haverkamp et al., 2016b), which was key to investigate odor learning with the proboscis. It is also naturally found in the nectar of *N. attenuata* (Kessler and Baldwin, 2007), the flower we used for our real flower investigation paradigm. The innate valence test was then followed by a training step, where the training odor was combined with a sugar reward (Fig. 3B), and a re-test, to investigate whether this experience increased the valence of the training odorant (Fig. 3C).

All three steps were conducted with the same moth on the same day with at least 30 min between trials. During the test phases, the odorant as well as clean air (mineral oil control) were supplied via two odor valves without obvious visual cues, spaced 45 cm apart at the upwind end of the wind tunnel. The valves were positioned about 20 cm from the upwind tunnel wall and mounted on metal poles 40 cm above ground. Linalool [here and in all other experiments: linalool (+/−), CAS 78-70-6; dilution: 10^−2^ in mineral oil, CAS 8042-47-5; loading volume: 10 μl] as well as solvent control (mineral oil, CAS 8042-47-5; loading volume: 10 μl) were pipetted onto small filter paper discs (diameter 1.3 cm, cut from a Rotilabo^®^ Rundfilter) and placed in 50 ml glass bottles (SCHOTT, Duran^®^), so the headspace could be collected. Charcoal-filtered air was used to push the headspace through Teflon tubing (diameter 4 mm) to the odor valves at an air flow speed of 0.05 l min^−1^. To exclude positional bias, the odor and control valve were switched between testing days. The time a moth spent at the odor valve minus the time spent at the control valve was used as a measurement of the odorant's valence. A significantly increased valence after the moth experienced the odor with the sugar reward (see below) would therefore indicate odor learning.

In the training paradigm, moths could perceive a training odor either with their antennae (and possibly their proboscis while approaching the flower) or with the proboscis only, while being rewarded with sugar solution (Fig. 3B). For the first experiment – to allow odor perception with the antennae – a small filter paper disc (diameter 1.3 cm; cut from a Rotilabo^®^ Rundfilter) was fixed with a metal clamp to the front of the training apparatus and was loaded with the training odor (Fig. 3B, upper panel). In the second experiment, we asked whether experiencing the training odor with the proboscis only is sufficient for odor learning. To prevent the moths’ antennae from coming into contact with the training odor during the experiment, the odor was confined to the inside of the artificial flower using an air push (in; air flow speed: 0.05 l min^−1^) and pull (out; air flow speed: 0.2 l min^−1^) system (Fig. 3B, lower panel). Similarly, as with the odor valves, the training odor was pipetted onto a small filter paper disc and placed into a 50 ml glass bottle, so the headspace could be collected and pushed through the proboscis tube within the artificial flower. As before, a moth was counted as ‘moths flown’ if it initiated flight during the first test. Moths that touched the flower at least once with their proboscis were counted as moths that ‘investigated flower’. In both experiments, a moth was considered trained when it had successfully foraged (‘successful’) at the artificial flower and had completely emptied the sugar reward. Only moths that met this training criterion were used for re-testing.

### Proboscis Y-maze paradigm

**Fig. 4.**
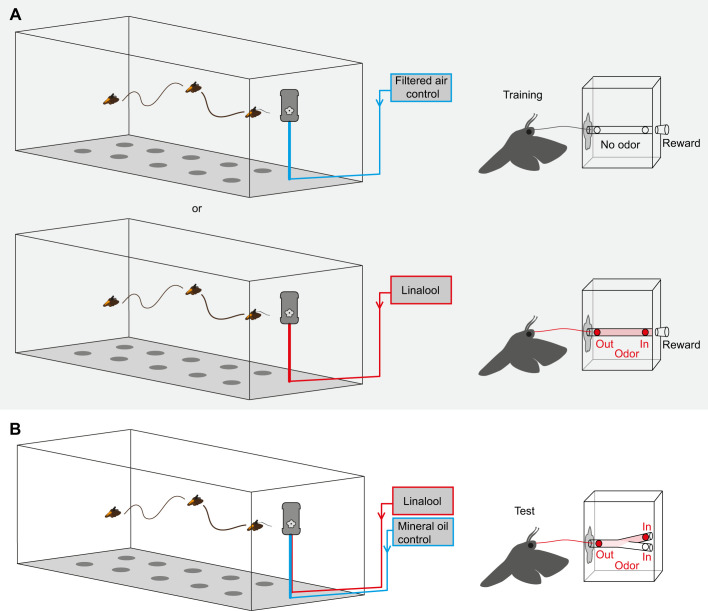
***Manduca* proboscis Y-maze paradigm – proboscis learning only.** (A) First, the moths were trained to investigate the artificial flower either in the absence (control group, top) or in the presence (experimental group, bottom) of the training odor. During training, the odor could only be perceived with the proboscis. (B) Moths were then tested with a proboscis Y-maze hidden within the artificial flower. One arm supplied the training odor (linalool), the other arm clean air (mineral oil control). Drawings not to scale.

**Fig. 5.**
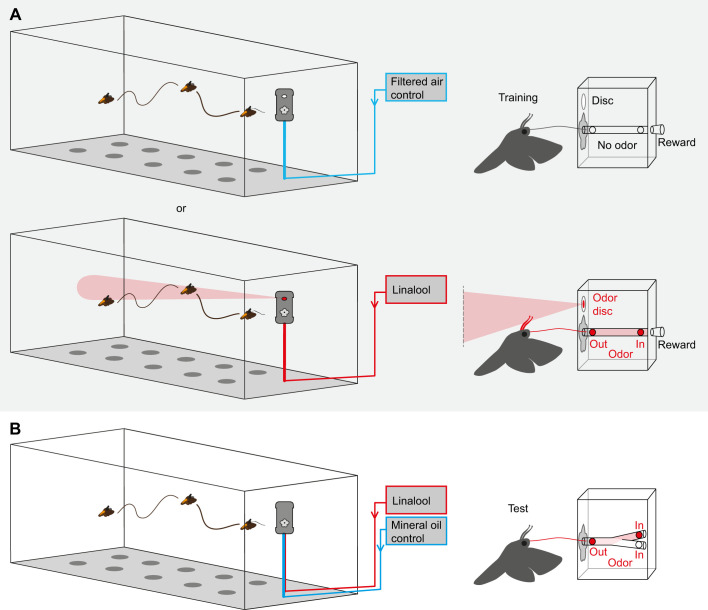
***Manduca* proboscis Y-maze paradigm – proboscis and antennal learning.** (A) Moths were trained to investigate the artificial flower either in the absence (control group, top) or in the presence (experimental group, bottom) of the training odor. During training, odor could be perceived with both the antennae and proboscis. (B) Moths were then tested with a proboscis Y-maze hidden within the artificial flower. As in the previous experiment, one arm supplied training odor (linalool) and the other arm clean air (mineral oil control). Drawings not to scale.

In a second paradigm, we asked whether the experience with a training odor would affect the performance of the proboscis within the flower (Figs 4 and 5). In the first experiment (Fig. 4), we wanted to know whether experiencing an odor with the proboscis only would influence the handling of a flower, i.e. the choice of the Y-maze arm within the flower. Therefore, we confined the odor to the inside of the artificial flower during training as well as during the test. As this is quite an artificial test situation – flower odor can usually already be smelled with the antennae during the approach and probing of the flower (Haverkamp et al., 2019) – we decided to conduct a second experiment and additionally added odor on the outside of our artificial flower during training (Fig. 5). We reasoned that this situation would be ecologically more relevant as moths use their antennae to locate a flower and use their proboscis to investigate the flower in more detail. During the test, however, odor was again confined to the Y-maze within the artificial flower.

For ease of the experiments we decided to simplify the process by using a control and an experimental group of moths. So, instead of comparing the performance of a given moth before and after training, we compared the performance of trained moths with that of other naive moths. During the training step, the control group was exposed to filtered air only while the experimental group was exposed to the training odor linalool. Again, a sugar reward was offered as an unconditioned stimulus to allow odor learning. This time, the moths were subsequently tested with a proboscis Y-maze within the artificial flower (Figs 2C, 4B and 5B). One of the Y-maze arms contained the mineral oil solvent control, the other arm the training odor (Fig. 2C). By adapting the airflow within the Y-maze, we ensured that only one arm of the Y-maze carried the odor (air removal speed: 0.6 l min^−1^). We recorded the first choice of the Y-maze arms and calculated the net time within the arm carrying the odor, i.e. (time in odor arm)−(time in control arm). Subsequently, we compared the control and experimental groups regarding their net time spent within the odor arm. As in the previous paradigm, a moth was counted as ‘moths flown’ if it initiated flight during the first test and as ‘investigated flower’ if it touched the flower at least once with its proboscis. Only moths that had successfully foraged at the artificial flower and emptied the sugar reward (‘successful’) were used for testing.

### Data analysis and statistics

The video data were analyzed manually using the software VLC media player 2.1.3 Rincewind. For the real flower investigation paradigm, the time point and success of the first 20 flower contacts were recorded and used for analysis. Insertion of the proboscis into the flower corolla was counted as ‘success’, and investigation of the flower without subsequent insertion of the proboscis as ‘no success’. In the case of success, the manipulation time until proboscis insertion (s) was recorded and the mean percentage success rate was calculated for the flower manipulations 1–5, 6–10, 11–15 and 16–20. For the odor choice paradigm time, the time spent at the control valve (s) and the time spent at the odor valve (s) were recorded before and after training. The net contact duration at the odor valve was calculated by subtracting the time spent at the control valve from the time spent at the odor valve. Similarly, the time spent in the control versus odor arm of the proboscis Y-maze was recorded and the net duration in the odor arm calculated for the proboscis Y-maze paradigm. Additionally, first choice and number of choices for each Y-maze arm were recorded for this paradigm and analyzed.

Statistical tests were performed using the statistics software GraphPad Prism version 7.05 for Windows (GraphPad Software, La Jolla, CA, USA, www.graphpad.com) or SPSS Statistics for Windows version 17.0 (SPSS Inc., Chicago, IL, USA). Data were tested for normality using the Shapiro–Wilk normality test and further analyzed with an appropriate parametric or non-parametric test.

## RESULTS

### Hawkmoths learn how to manipulate flowers successfully with the proboscis

**Fig. 6.**
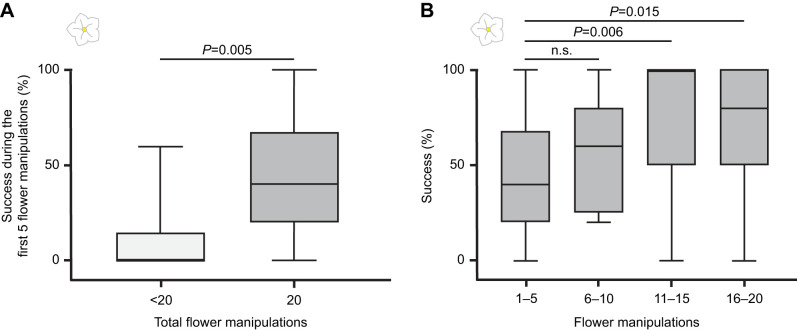
**Moths become more successful when investigating flowers.** (A) During the first five flower contacts, success rates differed between moths that had few (<20: *n*=8) and many (≥20: *n*=17) flower contacts. Moths that had higher success during the first five flower contacts tended to consecutively manipulate the flowers more often. Mann–Whitney *U*-test, *U*=22.5, *P*=0.005. (B) Feeding success increased with experience. When comparing the first five contacts with consecutive flower contacts, moths showed significantly higher success in later contacts (visits 11–15 and 16–20). Repeated measures ANOVA, *n*=17 moths, *F*_3,48_=6.136, *P*=0.001. Planned contrasts, 1–5 versus 6–10: *F*_1,16_=2.537, *P*=0.131; 1–5 versus 11–15: *F*_1,16_=10.112, *P*=0.006; 1–5 versus 16–20: *F*_1,16_=7.509, *P*=0.015. Box plots show the median, and first and third quartiles; whiskers show minimum and maximum values. White flower indicates real flower experiments.

In order to investigate whether the moths’ efficiency at natural flowers improves with experience and to create a learning baseline, we tested the moths using a *N. attenuata* flower array in the wind tunnel. Out of 107 ‘moths flown’, a total of 29 moths explored the flower array and touched at least one flower with their proboscis (‘investigated flower’). Of these, 31.0% had no success manipulating the flower, i.e. did not insert the proboscis into the flower opening; 51.7% had a success rate of ≥50%; and 20.7% had an overall success rate of ≥75%. Out of the 29 moths, 17 moths were highly motivated and had 20 (or more) flower contacts. When looking at the first five flower manipulations, the highly motivated animals exhibited a higher success rate than moths with fewer total flower contacts, suggesting that success increases the motivation to forage (Fig. 6A). Further analysis of moths with 20 flower contacts showed that they do learn to manipulate the flowers and become more successful over time (Fig. 6B). The shortest time of flower manipulation until proboscis insertion was less than 1 s, the maximum time 14 s. The average time before proboscis insertion was, however, 1 s. That means that the moths were generally quite quick to insert the proboscis into a non-moving *N. attenuata* flower at the given set-up.

### Hawkmoths learn odorants with their antennae, but not with their proboscis

**Fig. 7.**
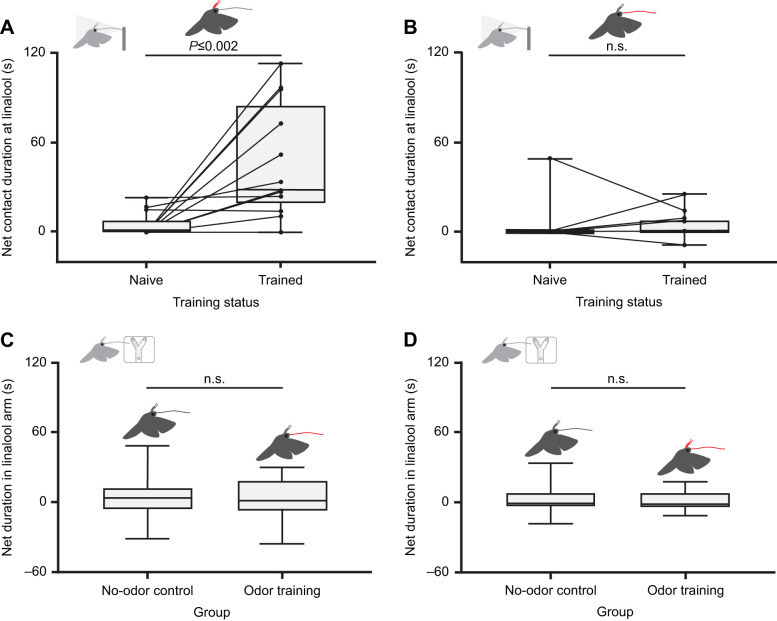
**Hawkmoths do not learn odors with their proboscis.** (A) Linalool attraction before and after antennal training. Wilcoxon matched-pairs signed rank test, *n*=13, *P*≤0.002. (B) Linalool attraction before and after proboscis training. Wilcoxon matched-pairs signed rank test, *n*=14, *P*>0.999. Moths experiencing linalool (+/−) with their proboscis (C) or antennae and proboscis (D) during training did not spend significantly more time in the linalool arm of the Y-maze than the control group. Unpaired *t*-test, C: *n*=20 per treatment, *t*_38_=0.329, *P*=0.744; D: *n*=15 per treatment, *t*_28_=0.317, *P*=0.753. Net contact duration at linalool (s): time spend at linalool (+/−) valve minus time spend at mineral oil control valve. Net duration in linalool arm (s): time spend in linalool (+/−) arm of Y-maze minus time spend in mineral oil control arm. Box plots show the median, and first and third quartiles; whiskers show minimum and maximum values; paired data of individual moths is visualized with connecting lines. Light gray moths: test paradigms; dark gray moths: training paradigms.

In the odor choice paradigm we tested whether *M. sexta* moths are able to learn odors with their antennae (moths flown: 46; investigated flower: 26; successful: 13; participated during re-test: 13) as well as their proboscis (moths flown: 28; investigated flower: 16; successful: 14; participated during re-test: 14) (Fig. 3). To assess whether our training odor (linalool) is innately neutral, we first established the valence of the training odor in a choice assay. As described previously by Bisch-Knaden et al. (2018), linalool was neither attractive nor aversive to naive moths as the moth spent the same amount of time at the two sources (Wilcoxon matched-pairs signed rank test, *n*=27, *P*=0.125). Hence, we were able to use it for our odor learning experiments. In a second step, i.e. the training step, the same moths were allowed to feed on sugar water from an artificial flower. During feeding, they could perceive linalool either by the antennae (and possibly the proboscis when approaching the flower) or by the proboscis only. After successful feeding, we again tested these moths for their preference for linalool in the aforementioned choice experiment. Moths that were allowed to perceive the odorant with their antennae during the training step readily learned the odorant and – in the subsequent test situation – spent significantly more time at the odor source than at the clean air source (mineral oil control) (Fig. 7A). In contrast, moths that were allowed to perceive the odorant during feeding with their proboscis only, later did not increase the time they spend at the odor source (Fig. 7B).

We next hypothesized that, although the odor experience via the proboscis does not affect the moths’ approach towards an odor source, it could still affect the subsequent navigation of the proboscis within the flower. We therefore designed a paradigm where again the moths were first trained to an odor. However, this time, the moths did not have to approach the odor during flight in the test situation. Instead, the moths were attracted to an artificial flower by visual cues and were asked to pinpoint the odor within a proboscis Y-maze hidden in the artificial flower (Figs 4 and 5). As mentioned before, for reasons of simplification, we used a control and an experimental group of moths for this paradigm. In the first experiment, odor was confined within the artificial flower during training (control: moths flown: 68; investigated flower: 50; successful: 29; participated during test: 20; experiment: moths flown: 37; investigated flower: 26; successful: 22; participated during test: 20). In the subsequent test, both the control animals as well as the moths of the test group inserted their proboscis an equal amount of time in both arms of the Y-maze (Fig. 7C). Also, both groups of moths made their first choice randomly (control: binomial test, *n*=20, *P*=0.824; test: binomial test, *n*=20, *P*=0.503) and did not choose one of the arms more frequently than the other (control: Wilcoxon matched-pairs signed rank test, *n*=20, *P*=0.850; test: Wilcoxon matched-pairs signed rank test, *n*=20, *P*=0.805), indicating that the odorant was not learned via the proboscis. We concluded that experiencing a flower odor with the proboscis alone affects neither the moths’ later approach to the odor in flight nor their proboscis navigation within the flower.

At the same time, moths that smelled linalool with their antennae while being rewarded with sugar water, later targeted linalool in flight. We, therefore, asked whether the experience of the odor via the antennae might influence the moths’ behavior with their proboscis, i.e. whether we would find information transfer from the antennae to the proboscis. In order to test this, we presented linalool to both the antennae and the proboscis, while the moth was feeding (Fig. 5A, bottom). For comparison, control moths were exposed to filtered air only during training (Fig. 5A, top). Afterwards, we again tested the navigation of the moths’ proboscis in the Y-maze (control: moths flown: 81; investigated flower: 34; successful: 22; participated during test: 15; experiment: moths flown: 69; investigated flower: 32; successful: 19; participated during test: 15) (Fig. 5B). Both the control and the experimental group investigated the two arms of the Y-maze for a similar amount of time (Fig. 7D), made their first choice randomly (control: binomial test, *n*=15, *P*>0.999; test: binomial test, *n*=15, *P*=0.607), and did not choose one of the arms more frequently than the other (control: paired *t*-test, *n*=15, *t*_14_=0.078, *P*=0.939; test: paired *t*-test, *n*=15, *t*_14_=0.345, *P*=0.735). Evidently, although moths seemed to be able to learn the odor of a rewarding flower and to follow such a flower plume afterwards, the performance of the proboscis within the flower was not affected by learning.

## DISCUSSION

Nectar-feeding insects such as bees, bumble bees, butterflies and moths are able to learn reliable food sources and return to flowers that promise high sugar rewards (Cnaani et al., 2006; Hill et al., 1997; Kandori and Yamaki, 2012; Kunze and Gumbert, 2001; Riffell et al., 2008). The specialization on one specific flower type can be of great advantage as it can save handling costs for the pollinator (Waser, 1986). Therefore, many insect species show flower constancy and keep visiting flowers of the same plant species that they have been successfully foraging at before (Amaya Márquez, 2009; Cunningham et al., 1998; Goulson and Cory, 1993; Hill et al., 1997). Among other cues, flower odors play an important role in the establishment of flower constancy (Gegear, 2005). So far, odor learning in moths has been assumed to function via the antennae only (Balkenius and Dacke, 2013; Cook et al., 2020; Cunningham et al., 2006, 2004; Daly et al., 2001a,b; Daly and Smith, 2000; Hartlieb, 1996; Ong and Stopfer, 2012; Riffell et al., 2008, 2013). However, hawkmoths (*M. sexta*) have olfactory sensilla not only on their antennae (i.e. their nose) but also on the tip of their proboscis, i.e. their tongue (Haverkamp et al., 2016b). Here, we asked whether hawkmoths become more efficient when feeding from flowers they have experienced before, and whether the olfactory sense on the proboscis is involved in this learning process.

To investigate whether hawkmoths learn from experience with a given flower type and create a baseline, we analyzed a moth's performance while consecutively feeding from several *N. attenuata* flowers provided in a flower array. Previous studies have shown that the feeding performance of *M. sexta* depends on the visited flower type or orientation (Campos et al., 2015; Haverkamp et al., 2019). However, we show that these hawkmoths, like butterflies (Kandori and Ohsaki, 1996) and bumble bees (Chittka and Thomson, 1997; Laverty, 1980, 1994; Laverty and Plowright, 1988), indeed become more efficient when repeatedly foraging from one flower type that emits an ecologically relevant odor, i.e. *N. attenuata* flowers (Fig. 6B).

From experiments with (artificial) flowers (Chittka and Thomson, 1997; Goyret, 2010; Goyret and Kelber, 2011; Goyret and Raguso, 2006; Kandori and Ohsaki, 1996), we know that learning of mechano-sensory as well as visual cues is involved in such experience-based improved feeding efficiency. We wanted to know whether olfactory learning with the proboscis plays an additional role in this learning process. To be able to compare antennal with proboscis learning, we first tested the hawkmoths using an antennal learning paradigm. As described before by Balkenius and Balkenius (2010), moths were able to learn the rewarding odorant within one trial and chose the odor source with the rewarding odorant significantly more often than the mineral oil control (Fig. 7A). However, when the moths were able to smell the odorant only with their proboscis during training, they did not navigate towards the odor source emitting the rewarding odorant in the consecutive test (Fig. 7B). We argued that this could either mean that the moths are not able to learn an odor with their proboscis or that moths are able to learn the odor with the proboscis, but do not use this information during the approach towards the flower. To find out whether hawkmoths use olfactory information learned with the proboscis while localizing nectar within the flower, we next tested the moths using a proboscis Y-maze. Again, we did not observe any learning. The moths spent equal amounts of time in the odor-emitting and the clean-air arm of the proboscis Y-maze after we trained them to the odorant (Fig. 7C). Given that encountering an odor with the proboscis alone is a rather artificial situation, we decided to do an additional experiment. In the wild, the moth can smell the odor of a flower even before inserting its proboscis in the flower corolla. That means that the odor can be considered a forward paired conditioned stimulus (CS) for the antennae as it will be present before the moth encounters the nectar (i.e. the unconditioned stimulus, US). In comparison, the proboscis might smell the odor for a much shorter time period when it is inserted into the flower. That means in terms of conditioning, this time span might be too short for the moth to create an association between odor (CS) and nectar (US) when using the proboscis to learn the CS. Still, would the moth be able to retrieve information via the proboscis that was acquired before by the antenna? In the desert ant *Cataglyphis fortis*, it was shown that the information about polarized light information acquired by one eye can later be retrieved by the other eye (Wehner and Müller, 1985). In dolphins such information transfer even happens between the visual and the acoustic sense (objects experienced by vision can later become recognized by echolocation and vice versa) (Pack and Herman, 1995). To test whether there is similar crosstalk between the moth's antennae and proboscis, we let the moth not only experience the odor via the proboscis inside the flower, but in addition already on the outside of the flower via the antennae (Fig. 5A). However, even this additional experience with the antennae did not affect the later performance of the moth's proboscis in the Y-maze (Fig. 7D). Hence, there does not seem to be any crosstalk between the antennae and proboscis taking place. We conclude that although *M. sexta* detects flower odors via both its antennae and the tip of the proboscis, only the antennae seem to be involved in the moth's olfactory learning. This might make sense from an ecological standpoint, as the antennae are used to find flowers at a distance. Hence, an early and correct decision, based on learning, might optimize energy gain. The proboscis ‘nose’, however, usually lies rolled up inside the moth head until the very last moment before flower contact. Although it stays unfurled during a foraging bout, a choice based on proboscis learning most likely would not yield a similar energy gain as hovering during the foraging bout is energetically costly.

So, what adaptive value could the olfactory sensilla on the tip of proboscis of the moth have? As Haverkamp et al. (2016b) suggested, these olfactory sensilla might be used to assess the quality of the flower. One could imagine a gustatory rather than an olfactory function as floral volatiles have been shown to be present in the nectar of some plant species (Kessler and Baldwin, 2007; Raguso, 2004). Also, appetitive volatile organic compounds in the nectar could increase the foraging motivation of the moth, similar to the way in which innately attractive benzyl acetone increases the foraging motivation in *M. sexta* when detected via the antennae (Haverkamp et al., 2016b). Contrary to our expectation, we did not find any increased foraging motivation when the moth perceived linalool (another ligand detected by the proboscis sensilla) with the proboscis. Single sensillum recordings by Haverkamp et al. (2016b) had shown that the multiporous sensillum styloconica responds to benzyl acetone with 62.6 spikes s^−1^ and to linalool with 22.5 spikes s^−1^. We therefore would have expected that there would be at least a baseline of attraction (maybe lower than that observed with benzyl acetone) and that learning would induce an increase in this baseline. However, we do not have any information on how the olfactory information of the proboscis is integrated in the brain of the moth and whether the difference in spike quantity is processed differently or translates in different ways to behavior. This of course raises the question of how the odor information of both ‘noses’ – the antennae and the proboscis – is processed in the moth's brain and how these pathways differ. It is known that odor information sensed by olfactory sensilla on the antenna of an insect is first processed by the antennal lobe and from there forwarded to higher brain centers such as the mushroom body and the lateral horn (Carey and Carlson, 2011). While the lateral horn codes for the innate valence of an odor, olfactory learning mainly takes place in the mushroom bodies (Carey and Carlson, 2011). It, therefore, will be interesting to test whether the olfactory information detected by the olfactory sensory neurons located on the tip of the proboscis is processed similarly or whether it is restricted to a local circuit innervating, for example, the subesophageal ganglion.

In summary, we suggest that the moth has two ‘noses’ dedicated to different tasks. The antennae are used for olfactory perception of the flower over distance. They are also important for learning to associate a flower odor with a nectar reward. The proboscis, however, seems to have a more limited function and might only be used for the assessment of flower quality.
